# Association Between Residential Rurality and Diagnostic Imaging Use in Pediatric Dog-Bite Encounters: A Cross-Sectional Multicenter Pediatric Health Information System Study (2021-2022)

**DOI:** 10.7759/cureus.94658

**Published:** 2025-10-15

**Authors:** Ryven E Mangundayao, Cory M Pfeifer

**Affiliations:** 1 College of Medicine, University of Arizona College of Medicine - Phoenix, Phoenix, USA; 2 Department of Radiology, Phoenix Children's Hospital, Phoenix, USA

**Keywords:** animal bite, dog bites, imaging, pediatrics, rural, urban

## Abstract

Introduction: Imaging helps evaluate pediatric dog-bite injuries, but utilization may vary by rural-urban setting. We aimed to determine whether rural-urban commuting area (RUCA) classification of residence is associated with diagnostic imaging utilization in pediatric dog-bite encounters at US children’s hospitals.

Methods: We conducted a retrospective cross-sectional study using the Pediatric Health Information System (PHIS) across 45 children’s hospitals (January 2021 to December 2022). Patients aged ≤18 years with the International Classification of Diseases, 10th Revision (ICD-10) dog-bite codes (W54.0XXA; S01/S41/S61/S71 series) were included, and those with invalid RUCA codes were excluded. The exposure was RUCA code (2020 scale: 1 urban to 10 rural). The primary outcome was imaging during the encounter, defined as radiography, ultrasonography, computed tomography, or magnetic resonance imaging. Multivariable logistic regression modeled RUCA as a continuous predictor of imaging while adjusting for sex, payer, disposition, and length of stay (LOS). Model-predicted probabilities were summarized across one-unit RUCA bands, and linear regression was used to assess trends. PHIS data were de-identified.

Results: Among 13,901 encounters, the median age was seven years (interquartile range: 3-11), and 57.1% were male. Imaging occurred in 2,992 encounters (21.5%). Unadjusted imaging rates were urban 21.2% vs. rural 26.5%, an absolute difference of 5.32 percentage points (95% confidence interval (CI): 2.37-8.27; p < 0.001). The predicted probability of imaging increased stepwise with rurality; linear regression of RUCA bands showed a positive association (R² = 0.91; F(1, 8) = 81.97; p < 0.001). In the adjusted analysis, a higher RUCA code was associated with greater odds of imaging (β = 0.052 per RUCA point; odds ratio (OR) ≈ 1.05 per point; z = 3.37; p < 0.001), corresponding to approximately 8.61 percentage points higher absolute imaging between the most urban and most rural groups. A longer LOS was also associated with imaging (β = 0.341 per day; OR ≈ 1.41 per day; z = 9.37; p < 0.001). Most patients were discharged from the emergency department (83.0%), and 5.0% were admitted.

Conclusion: Rural residence was associated with higher imaging use in pediatric dog-bite encounters at children’s hospitals. These observational findings are hypothesis-generating; differences may reflect practice patterns, case-mix, or selection rather than causation. Absolute differences were modest, suggesting system-level relevance but limited impact for most individual encounters.

## Introduction

Geographic disparities in pediatric trauma care are well documented, with children in rural areas experiencing higher injury rates and worse outcomes than their urban counterparts [1]. For example, unintentional injury rates among rural children are over 50% higher than those in urban settings, and injury-related mortality is nearly double [1]. Contributing factors include limited access to specialized pediatric care in rural regions and longer travel distances to trauma centers, which can result in delayed treatment [1]. Millions of dog-bite injuries occur annually in the United States, and children comprise a disproportionately affected group [2]. Approximately 40% of all pediatric trauma cases are due to dog bites, and more than half of US children will sustain a dog bite by 12 years of age [2]. While most bites are minor, some lead to serious infections, disfigurement, or even death, making dog bites a notable public health concern [3]. Notably, most published studies on pediatric dog bites have originated from urban trauma centers, with little attention to rural cases [3]. Emerging data suggest that rural pediatric dog-bite victims may have distinct characteristics and care courses: a statewide analysis found that children from rural areas tended to be younger and more often from low-income communities, and they were more likely to experience delays in receiving care [3]. That population-based study identified rural children as a previously underrecognized vulnerable group in the context of dog-bite injuries [3].

In evaluating pediatric dog-bite injuries, imaging (e.g., radiographs or computed tomography (CT) scans) is often used to detect fractures or foreign bodies; however, evidence indicates that this practice is frequently of low yield [2]. In one institutional series, only about 11% of imaging studies performed for pediatric dog-bite wounds identified a relevant injury, suggesting that imaging was overutilized in the majority of cases [2]. Even modest geographic differences in imaging can translate into a nontrivial radiation burden at the population level. A typical head CT delivers an effective dose of approximately 2 mSv, roughly 100 times that of a posteroanterior chest radiograph. Therefore, a five-percentage-point difference in imaging across 10,000 similar encounters would equate to ~500 additional CT examinations and ~1 Sv of cumulative population dose [4]. Epidemiologic studies have demonstrated small but measurable increases in subsequent malignancy after childhood CT exposure, underscoring the importance of minimizing low-yield imaging [5]. Unnecessary imaging is a particular concern in children, who are more sensitive to ionizing radiation and face greater cumulative lifetime risk from exposure [2]. However, there is currently no consensus or established guidelines on when imaging is truly warranted for pediatric dog-bite injuries, and little published data exist to inform best practices [2]. For instance, a recent survey of trauma providers found that those in rural hospitals, who often have lower pediatric volumes and fewer pediatric specialists, reported using broad imaging (e.g., whole-body CT scans) more frequently in injured children when standardized pediatric trauma protocols were lacking [6].

The rural-urban commuting area (RUCA) code system provides a detailed classification of community rurality based on population density and commuting flow data [7]. RUCA codes delineate rural and urban areas at the census tract level, offering a nuanced sub-county categorization that captures gradients of urbanization beyond simple county designations [7]. The primary objective of this study was to explore whether residence rurality, measured by RUCA code, is associated with the likelihood of diagnostic imaging use in pediatric dog-bite encounters at US children’s hospitals; this retrospective cross-sectional analysis is hypothesis-generating and not designed to infer causality.

## Materials and methods

This study used a cross-sectional analysis of pediatric dog-bite injuries using a national hospital database. We retrospectively analyzed encounters between January 2021 and December 2022. As the data were de-identified and publicly available, the study was deemed exempt from Institutional Review Board (IRB) approval. All work complied with the Health Insurance Portability and Accountability Act (HIPAA); no individually identifiable health information was accessed.

Data source and setting

This study was conducted using the Pediatric Health Information System (PHIS), an administrative database of pediatric hospital encounters maintained by the Children’s Hospital Association. PHIS collects clinical and resource utilization data from inpatient, emergency department (ED), observation, and ambulatory surgery encounters at over 45 children’s hospitals across the United States [8]. This multi-institutional data source provided a broad geographic representation of pediatric dog-bite cases.

Patient selection

We included all pediatric patient encounters (age ≤18 years) in the PHIS database during the study period (2021-2022) that involved documented dog-bite injuries. Dog-bite encounters were identified by the International Classification of Diseases, 10th Revision (ICD-10) diagnosis codes [9]. Specifically, we included encounters with a primary diagnosis code of W54.0XXA (“bitten by dog, initial encounter”) or with primary injury diagnoses in the S01 (head), S41 (arm/shoulder), S61 (wrist/hand), or S71 (leg) series indicating dog-bite wounds [9]. Those not meeting these criteria or with patients aged over 18 years were excluded. We also excluded records missing a valid RUCA code for the patient’s residence (−1). After applying these criteria, 13,901 encounters were included in the final analysis.

Variables and definitions

**Figure 1:**
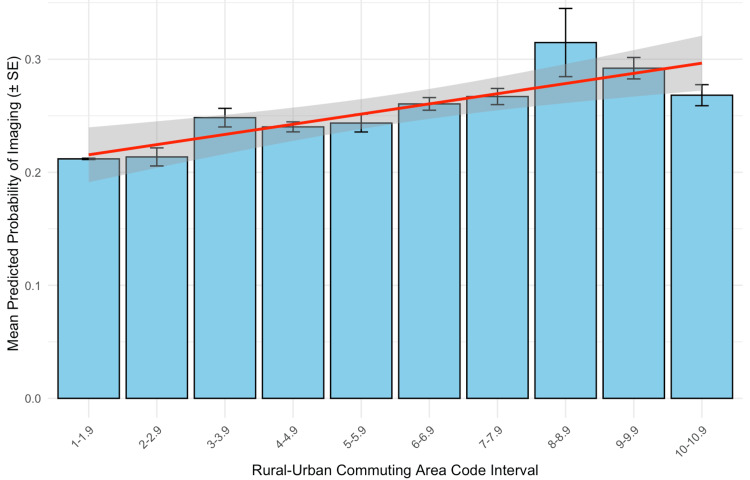
Mean model-predicted probability of imaging by rural-urban commuting area (RUCA) interval Bars depict the mean predicted probability of imaging for encounters grouped by RUCA code intervals (1–1.9, 2–2.9, …), with error bars showing the standard error. The fitted linear regression line (red) with 95% confidence band (gray) indicates an increasing probability of imaging with greater rurality (R² = 0.91; F(1, 8) = 81.97; p < 0.001). Statistical significance was defined as p < 0.05 (two-sided).

The primary exposure variable was the rurality of the patient’s residence, which was categorized using the RUCA code. RUCA codes (2020 classification) range from 1 (metropolitan urban core) to 10 (rural area) and classify US census tracts by degree of urbanization and commuting patterns (Table 1) [7]. For analysis, we specified RUCA as a linear continuous predictor to preserve ordinality and statistical power, given sparse counts at higher codes and the near-monotonic band-level trend in Figure 1, and dichotomized RUCA into "urban" (codes 1-3) vs. "rural" (codes 4-10) categories for subgroup comparisons. Additional variables collected as potential confounders included patient sex, insurance type (e.g., public vs. private insurance), hospital disposition (e.g., discharged home vs. admitted or transferred), and length of stay (LOS) (days).

**Table 1:** Primary RUCA codes (2020) Note: UA = urban area; primary flow = largest commuting flow from census tract; RUCA: rural-urban commuting area Source: US Department of Agriculture, Economic Research Service [7]

Code	Classification description
1	Metropolitan core: primary flow is within an urban area of 50,000 or more people (metro UA)
2	Metropolitan high commuting: primary flow is 30 percent or more to a metro UA
3	Metropolitan low commuting: primary flow is 10 percent to 30 percent to a metro UA
4	Micropolitan core: primary flow is within an urban area of 10,000 to 49,999 people (micro UA)
5	Micropolitan high commuting: primary flow is 30 percent or more to a micro UA
6	Micropolitan low commuting: primary flow is 10 percent to 30 percent to a micro UA
7	Small town core: primary flow is within an urban area of 9,999 or fewer people (small town UA)
8	Small town high commuting: primary flow is 30 percent or more to a small town UA
9	Small town low commuting: primary flow is 10 percent to 30 percent to a small town UA
10	Rural area: primary flow is to a tract outside an UA

Outcome

The primary outcome variable was imaging utilization, defined as a binary indicator of whether any diagnostic imaging study (i.e., radiography, ultrasonography, CT, or magnetic resonance imaging) was performed during the encounter. PHIS does not uniformly capture imaging modality across contributing sites; consequently, modality-specific analyses were not performed, and the outcome was analyzed as a binary “any imaging” indicator.

Data sources and measurements

All data were obtained from the PHIS database, which provides de-identified patient-level information (demographics, diagnoses, procedures, and outcomes) [8]. Each patient’s residence was mapped to a RUCA code using the 2020 classification from the US Department of Agriculture’s Economic Research Service [7]. Imaging utilization was derived from PHIS procedure/charge records; because modality-specific Current Procedural Terminology (CPT) reporting is not uniform across hospitals, radiology services were identified from site-standardized radiology charge/transaction classes, and the outcome was operationalized as a binary indicator of “any imaging” (yes if ≥1 study; no otherwise).

Variable coding and missing data

Sex was coded as female, male, or unknown. Payer and disposition categories with sparse counts were collapsed for modeling stability (see "Statistical analysis" for the collapsing approach). RUCA and LOS were modeled as continuous variables. Data cleaning steps included restricting the analysis to patients aged ≤18 years, excluding encounters with invalid/missing RUCA codes (−1), and removing fields with more than 50% missingness prior to analysis. Multivariable models used listwise deletion (no imputation). The proportion with invalid/NA RUCA is reported in "Results," and covariate missingness for modeling variables was low. Exact factor levels and coding are available on request to support reproducibility.

Comparison strategy

This study compared imaging utilization in children from rural and urban areas. We stratified the included encounters by RUCA-based residence category (rural vs. urban) to examine unadjusted differences in imaging rates. No separate control group was employed; instead, all eligible patient encounters were analyzed, with rural vs. urban subgroups serving as the comparison groups. This approach enabled us to assess the association of rurality with imaging use while controlling for other variables in multivariable analyses.

Statistical analysis

**Figure 2:**
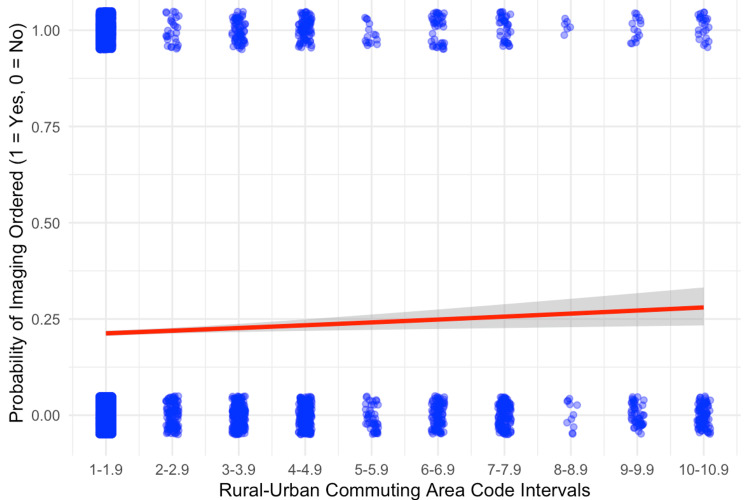
Imaging utilization in relation to rural-urban commuting area (RUCA) codes Each blue jitter point represents an individual encounter (1 = imaged, 0 = not imaged). The red line shows the fitted logistic regression curve, with a 95% confidence interval (gray). Higher RUCA scores indicating greater rurality were linked to an increased likelihood of imaging (β = 0.052 per RUCA point; odds ratio (OR) ≈ 1.05 per point; z = 3.37; p < 0.001). Statistical significance was defined as p < 0.05 (two-sided).

Encounters were first grouped into 10 one-unit RUCA intervals (1-1.9, 2-2.9, …, 10-10.9). For each interval, we calculated the mean model-predicted probability of imaging from the multivariable logistic model and displayed it with its standard error as bars (Figure 1). To test the overall trend in these interval-level means, we fitted an ordinary least-squares linear regression across RUCA intervals and reported the coefficient of determination (R²) and the F-test used to obtain the two-sided p-value. For unadjusted rural-urban comparisons, a two-sample test of proportions was used to estimate the absolute difference in imaging rates with 95% confidence intervals (CIs) and corresponding p-values. At the encounter level, we then evaluated the association between rurality and imaging with a multivariable logistic regression in which imaging utilization (1 = imaged, 0 = not imaged) was the dependent variable and RUCA code (continuous) was the primary exposure (Figure 2); LOS (days) was included as a covariate. To mitigate quasi-separation from sparse categories, rare factor levels (payer, disposition) were prospectively collapsed into an "Other/rare" category, and disposition was dichotomized for modeling stability ("Discharge to home or self-care" vs. "Other/rare"). Reference levels were set a priori. LOS was treated as a linear term in days; an exploratory log transformation (log1p) produced similar results, so the linear specification was retained for interpretability. From this model, we reported regression coefficients (β), adjusted odds ratios (OR = e^β) with 95% CIs, and the corresponding Wald z tests used to obtain two-sided p-values. Corresponding test statistics were reported, along with two-sided p-values, in "Results" and figure captions. Sensitivity analyses were performed by refitting the logistic model with RUCA specified as 10 categorical intervals (reference: 1-1.9) and by repeating the model after removing LOS. Effect estimates and inferences for RUCA were materially unchanged, indicating no material departure from linearity and supporting the retention of the linear RUCA term. Because disposition and LOS could lie on the causal pathway between rurality and imaging, adjusted estimates were interpreted with caution to avoid over-interpretation due to potential over-adjustment. Model diagnostics included variance inflation factors to screen for multicollinearity and a Hosmer-Lemeshow goodness-of-fit check for calibration. Invalid RUCA values (−1) and records with missing modeling fields were excluded (complete-case analysis). All statistical analyses were conducted in RStudio (v2024.12.0+467; RStudio Team, Boston, MA) using Tidyverse for data processing; statistical significance was defined as a two-sided p-value < 0.05. Figures were produced with base ggplot2 (v3.5.0).

## Results

Patient characteristics

A total of 13,901 pediatric encounters for dog-bite injuries were identified across 45 US children’s hospitals (2021-2022). The median age was seven years (interquartile range: 3-11); 7,942/13,901 (57.1%) patients were male, and sex was missing for seven patients. The RUCA codes span from 1 to 10. Imaging was performed in 2,992 of 13,901 (21.5%) encounters. Across all encounters prior to RUCA exclusion (n = 14,117), valid RUCA was available for 13,901 (98.5%); 216 (1.5%) had invalid/NA RUCA and were excluded. Unadjusted imaging rates were 21.2% in urban encounters (RUCA: 1-3; 2,717/12,843) vs. 26.5% in rural encounters (RUCA: 4-10; 251/948), an absolute difference of 5.32 percentage points (95% CI: 2.37-8.27; p < 0.001). Disposition included 11,535/13,901 (83.0%) ED discharges and 688/13,901 (5.0%) inpatient admissions; the remainder were 672/13,901 (4.8%) observations, 519/13,901 (3.7%) clinics, 375/13,901 (2.7%) ambulatory surgeries, and 112/13,901 (0.8%) classified as all other encounter types.

Imaging by rurality

When stratified by the RUCA classification, the mean model-predicted probability of imaging increased from the most urban to the most rural bands (Figure 1). An ordinary least squares (OLS) trend test across RUCA bands showed a strong positive association (R² = 0.91; F(1, 8) = 81.97; p < 0.001).

Multivariable analysis

In the logistic regression model, a higher RUCA code was independently associated with greater odds of imaging (β = 0.052 per RUCA point; OR ≈ 1.05 per point; z = 3.37; p < 0.001) (Figure 2). Thus, each one-point increase in the RUCA code corresponded to a higher likelihood of an imaging study being performed. Marginal standardization indicated adjusted probabilities of 21.14% at RUCA 1 vs. 29.75% at RUCA 10, an adjusted absolute difference of 8.61 percentage points. Although statistically significant, the adjusted effect size was small at the encounter level; even across a large rurality gradient (e.g., RUCA: 1-10), the model implies a roughly 1.4-fold difference in the odds of imaging. These findings should be interpreted in the context of unmeasured clinical factors not available in PHIS (e.g., injury site, severity, provoked vs. unprovoked mechanism, animal breed, and wound depth), which could confound the association.

Length of stay

The LOS was positively associated with imaging. In the multivariable logistic model, each additional hospital day increased the odds of imaging (β = 0.341 per day; OR ≈ 1.41 per day; z = 9.37; p < 0.001).

Heterogeneity and site-level consistency (exploratory)

Age

Unadjusted rural-urban absolute differences in imaging were 6.98 pp for ages 0-5 (24.8% vs. 17.9%), 6.24 pp for ages 6-12 (27.3% vs. 21.1%), and 2.10 pp for ages 13-18 (31.6% vs. 29.5%). A likelihood-ratio test of interaction (RUCA × age group) was significant (ΔDeviance = 119.21 on 4 df; p < 2.2×10⁻¹⁶), suggesting effect heterogeneity by age.

Payer

Unadjusted rural-urban absolute differences varied across payer categories (e.g., commercial health maintenance organization 11.6 pp; Medicaid-managed care 3.3 pp), but a RUCA × payer interaction test was not significant (ΔDeviance = 10.62 on 11 df; p = 0.476).

Sites

Across hospitals with both rural and urban encounters, the rural-urban difference in imaging was directionally positive (rural > urban) in 23 of 42 sites.

## Discussion

This multicenter analysis showed that residence rurality, measured by RUCA, was independently associated with imaging use for pediatric dog-bite encounters. Rural patients were statistically more likely to undergo imaging for dog-bite wounds (p < 0.05), whereas urban patients were imaged more selectively. The magnitude of this disparity was modest in absolute terms, suggesting that, although it is statistically significant, its clinical relevance should be interpreted with caution, and residual confounding by unmeasured clinical factors (e.g., wound severity, mechanism, and local practice norms) may partly account for the observed gradient. Critically, because we lacked direct measures of injury severity or explicit imaging indications, case-mix differences could entirely account for the rural-urban gradient; therefore, causal inference about practice disparities is not appropriate.

Consistent with this, the per-point adjusted effect was modest, and even the cumulative gradient across the rural-urban spectrum suggests only a moderate increase in odds, indicating limited clinical impact for most individual encounters. It is plausible that rural clinicians employ precautionary imaging due to limited on-site specialty support or concerns about missing occult injuries. In contrast, urban pediatric centers with immediate access to surgical specialists may rely more on clinical examination and ordering imaging only when strongly indicated. Thus, the rural-urban gap in imaging should be interpreted as a hypothesis-generating signal that may reflect differences in resource availability and practice patterns; alternative explanations, such as greater injury severity among rural presentations or selective transfer patterns to children’s hospitals, are also plausible and were not directly measured.

Clinically, the slightly higher imaging rate in rural cases may result in additional radiation and costs for some children; however, this may also provide reassurance in settings where follow-up is uncertain. Distinguishing true clinical benefits from overutilization is essential, as a statistically significant variation is not inherently synonymous with meaningful improvement in patient outcomes. Here, the observed difference may indicate a practice disparity more than a performance disparity, warranting attention to ensure that imaging is used optimally across all settings. Accordingly, the results reflect a statistically robust gradient with a modest absolute effect, which may be more relevant for system-level planning and rural readiness than for changing imaging decisions in otherwise typical individual cases.

Exploratory analyses suggested that rural-urban differences were larger in younger children (0-12 years) and attenuated in adolescents, were not modified by payer type, and were directionally consistent in a slight majority of sites (rural > urban in 23 of 42 hospitals with both groups). These patterns provide context for where practice variation may be most pronounced and may help target guideline dissemination and readiness efforts.

As previously mentioned, research suggests that imaging in pediatric dog-bite care is often overused [2]. Furthermore, imaging practices differ by hospital type: pediatric trauma centers tend to follow more stringent criteria for scans, whereas general (often non-pediatric) centers have been shown to image more liberally. A recent meta-analysis of blunt trauma care found that children evaluated at adult/community trauma centers underwent significantly more CT scans (for head, abdomen, spine, etc.) than those at pediatric centers, presumably due to differing expertise and protocols [10]. This pattern aligns with the present study’s observation that rural children, who are often initially seen in non-pediatric facilities, have higher imaging rates, potentially reflecting a lower threshold for obtaining studies in those environments. Current clinical recommendations emphasize that imaging for animal bites should be guided by suspicion of deep injury. Radiographs or CT scans are indicated primarily when there is concern for bone involvement, joint penetration, or a retained tooth fragment, rather than as routine [11]. The present study’s results suggest that urban centers may be closer to this ideal, whereas some rural settings might err on the side of caution with extra imaging. It is essential to note that, while such caution can detect occult injuries, it must be balanced against the risks and costs associated with low-yield studies. In practical terms, the present study highlights the need for consistent guidelines applicable to both rural and urban hospitals to assist clinicians in determining when imaging is truly warranted in pediatric dog-bite cases. The results of this study add to the growing body of literature on rural-urban disparities in pediatric trauma care. Massand et al. analyzed over 1,000 pediatric dog-bite cases in a statewide cohort and identified rural children as a particularly vulnerable group, being, on average, younger and more likely to experience delayed care or require inter-hospital transfer than urban children [3]. Those findings are consistent with the present study’s observation that rural patients often undergo a different initial workup (e.g., more imaging, likely as part of preparation for possible transfer). Similarly, an English national analysis of hospital episode data found higher dog-bite admission rates in rural areas than in urban areas, with substantial geographic variation [12]. Notably, the overall rise in admissions over time was driven by adults rather than children, and the study did not determine the reasons for the rural-urban difference [12]. Moreover, children living in rural regions generally have a higher overall risk of injury. For instance, an analysis of Oregon’s trauma system found that pediatric injury incidence and mortality were significantly greater for rural children, the annual rate of traumatic brain injury in rural youth was ~50% higher than that in urban youth, and rural children had more than double the odds of trauma mortality even after adjusting for injury severity [13]. Such disparities are thought to stem from longer emergency response times, fewer pediatric-trained providers, and delayed definitive care [13,14]. The findings of increased imaging utilization in rural dog-bite cases could be viewed as an extension of these disparities, indicating that rural providers may compensate for limited resources by using more diagnostics. Notably, this practice difference aligns with documented patterns of greater emergency department utilization in rural communities [15]. However, more intervention does not inherently yield better outcomes; validated pediatric head-trauma decision rules with near-100% sensitivity for clinically important brain injury enable safe reductions in CT use without increasing missed injuries [16]. The challenge, as highlighted by pediatric-readiness policy statements, is to ensure that children in all settings, including resource-limited rural EDs, receive timely, effective trauma care while avoiding overtreatment [17]. In essence, the results of the present study corroborate prior evidence of rural-urban differences and emphasize the importance of targeted improvements in rural pediatric trauma management.

Limitations

The limitations of this study include its retrospective and observational nature, which preclude the determination of causation and are subject to residual confounding. Because PHIS captures encounters only at participating children’s hospitals, encounters at community hospitals and adult trauma centers are not represented. This sampling frame may preferentially include rural children who are transferred or otherwise more complex, introducing selection bias that could overestimate imaging in rural settings relative to population-wide practice and limiting generalizability. We relied on an administrative database and RUCA classifications to define rural vs. urban status; however, such data may misclassify some areas or fail to capture nuances such as the distance to a pediatric trauma center. The data source lacked clinical granularity in terms of wound severity, imaging indications, and outcomes. For example, we could not discern whether imaging was performed due to specific concerns (e.g., suspected fracture or joint involvement) or as a general precaution. Key clinical details, such as injury site and depth, bite mechanism (provoked vs. unprovoked), and animal/breed information, were unavailable, limiting the adjustment for potential confounding by severity and indication for imaging. As a result, severity- and indication-related case-mix differences could fully explain the observed association, and the present study cannot distinguish between practice variation, case severity, or selection effects. Including LOS and disposition may also introduce over-adjustment if these variables lie on the causal pathway between rurality and imaging; accordingly, adjusted estimates are interpreted with caution. The findings should be regarded as hypothesis-generating only. Additionally, the study’s outcome was imaging utilization itself, rather than patient-oriented outcomes. We did not directly assess whether higher or lower imaging rates affected complication rates, missed injuries, LOS, or costs, which may have clinical implications. There is also a potential selection bias: rural injuries that were minor might not have presented to hospitals at all, whereas all major injuries likely did, which could skew comparisons if urban centers see a broader spectrum of minor dog bites. The missingness of RUCA codes was low (1.5%), which reduces the likelihood of bias from differential RUCA availability; however, exclusions for invalid/NA RUCA codes may still limit representativeness in some subregions. Generalizability is another concern; the findings may not apply to pediatric dog-bite incidents in other contexts or regions with different healthcare infrastructures. Finally, because we grouped diverse rural and urban settings, we may have obscured intra-group variability (e.g., differences between small remote clinics and larger rural hospitals). These limitations underscore the need to interpret the study’s conclusions as associations rather than causal effects and to consider them as hypothesis-generating for future prospective research.

## Conclusions

Residence rurality was associated with higher imaging use in pediatric dog-bite encounters, though the adjusted effect per RUCA point was modest, suggesting limited impact at the individual-encounter level but relevance for system planning. Given the observational design, lack of injury-severity data, and potential selection biases, these results should be interpreted as hypothesis-generating rather than directive for practice change. Future work should (i) incorporate detailed clinical severity and imaging indication data across children’s and community hospitals to assess case-mix differences; (ii) evaluate heterogeneity across patient subgroups and sites using prespecified interaction analyses; (iii) link imaging patterns to patient-centered outcomes, radiation exposure, downstream testing, and costs; and (iv) test, in prospective or quasi-experimental studies, whether standardized imaging criteria or decision support meaningfully reduce low-yield imaging without compromising safety. Such studies are needed before specific practice recommendations can be made.
